# Chlorosis: Its Complications and Treatment

**Published:** 1898-10-22

**Authors:** C. Y. Biss


### Medical Progress and Hospital Clinics.

### CHLOROSIS: ITS COMPLICATIONS AND TREATMENT.

Abridged Report of a Clinical Lecture delivered at the Middlesex Hospital by C. Y. Biss, M.D., F.R.C.P.

[Specially Reported for The Hospital, and Corrected by the Author.]

It was pointed out that chlorosis, though so common a disease, is a malady which often entails considerable danger, and may be followed by grave complications. The condition which most resembles it is the anæmia which often ushers in tuberculosis; and it is very important to distinguish it from this. The complications are:—

1. **General and Nervous Debility.**—The subjects of chlorosis suffer from lassitude, muscular weakness, headaches, &c., and as this occurs usually between the ages of 15 and 20, and principally in the female sex, it may be—as Fagge points out—the starting point of phthisis, hysteria, chorea, gastric ulcer, and exophthalmic goitre. Slighter disorders, as migraine, feeble digestion, and neurasthenia, may also follow. In this way the foundation of permanent ill-health may be laid.

2. **Cardiac Dilatation.**—This is a common and grave complication, and is partly the cause of the breathlessness on slight exertion so often found. The enfeeblement of the cardiac walls and consequent weakness of the circulation are also causes of thrombosis. It is usually recoverable from, but when neglected or when it recurs permanent changes in the heart may take place.

3. **Thrombosis.**—This is an occasional, and may be a fatal complication. The formation of thrombi is always to be dreaded when the blood is poor and the circulation feeble, as in some of the later stages of cancer, phthisis, and in some of the specific fevers. A case related to illustrate this was that of a girl admitted into hospital in a semi-comatose condition, and with a slight rise of temperature. She had no other symptoms, and no physical signs except those of chlorosis. Death occurred on the second day after admission, and clots were found in all the cerebral sinuses, to which cause her death was wholly due.

4. **Ulceration of Stomach.**—It is probable that the two main causes of these ulcers are—(1) Lowered vitality of the tissue; (2) the formation of a small thrombus at some point of the submucosa, the membrane then undergoing necrosis, becomes gradually eroded by the gastric juice, and hollowed out into a small ulcer. Now the earlier a gastric ulcer is detected, or even suspected, the earlier the patient can be put under suitable treatment; and the earlier treatment is begun the more likely it is to be effective; but if the probability of ulcer be overlooked, and the symptoms vaguely referred to dyspepsia, treatment will be unintelligent and ineffective, and the ulcer may pass into a chronic form, or even perforate into the peritoneal cavity.

5. **Gastralgia.**—Cases of gastric neuralgia without any organic lesion do occur, and, as they are difficult to distinguish from those of ulceration, it is better in doubtful cases to treat the case as one of gastric ulcer. A case was related of a girl, age 18, who complained of palpitation and severe pain at the epigastrium and in the left side; she was intensely anæmic, and had cardiac dilatation, but only slight tenderness on pressure over the epigastrium. The medical man who brought her remarked, "She has had almost all the drugs in the Pharmacopœia, without benefit." She had not, however, been treated by that invaluable medicine—rest, so she was put to bed for a fortnight, kept upon a milk diet, and given carbonate of iron in a pill form. From that day she had no more pain, and made a rapid recovery. It is difficult to believe that an ulcer was present here, and the characters of the pain pointed rather to gastric neuralgia than to ulcer.

6. **Dilatation of Stomach.**—This is an occasional result of chlorosis, and it is probably always either associated with, or resulting from, the presence of gastric ulcer.

The therapeutical measures may be summarised under the following heads:—

1. **Rest.**—It was pointed out that rest is the most important curative agency we possess, and that it is required in all cases of chlorosis, and that when gastric ulceration is also present then the rest must be understood to include *gastric rest*, and in the worst cases complete rest for the stomach for a few days, during which rectal feeding should be carried on, while in the slighter cases a fluid diet should be administered in small quantities at appropriate intervals.

A case was quoted of a woman, aged 26, who gave a history of having been "pale" for six years. Her breath was short; she had palpitation, indigestion, constipation, amenorrhœa, morning retching, and swelling of the legs and feet. She had been treated with various drugs, but without definite improvement, and was so weak that she could scarcely walk. She was sent home and kept in bed for several weeks, the bed being placed during the daytime near an open window, all excitement was avoided, regular action of the bowels secured, and she was given pills containing carbonate of iron. In four months she completely recovered, and has remained well ever since.

2. **Aperients.**—Aperients are needed, especially when the patient is kept at rest—such as tabloids of cascara sagrada, compound liquorice powder, &c. If there has been protracted constipation the large intestine should be emptied at the commencement by two or three doses of a saline purgative.

3. **Feeding.**—A chlorotic patient should be abundantly fed, but not at the beginning of the treatment, or if there is any suspicion of gastric ulcer. Feeding is best commenced by fluid nourishment, followed by eggs beaten up in milk, custard and milk puddings, and so on; and as soon as it is certain that the digestion will bear it, it is well to feed very liberally. While the patient remains in bed massage may be employed.

4. **Iron.**—Iron is the best specific for this disease; but two conditions, it was pointed out, must be observed in this administration—first, that one of the milder preparations, such as the carbonate, be selected; second, that it should be given in the form of pills, palatinoids, jelloids, or tabloids, which are not likely to be disintegrated till they reach the intestine. If these two points are attended to, iron may be given even in cases complicated with gastric ulcer. Another essential point is to give the iron in sufficiently large doses; the lecturer's own practice being to give three or four Pilulæ Ferri thrice daily, after food, or at other convenient intervals. Liquid preparations of iron, especially the more strongly astringent ones, should not be given at all, as they tend to irritate the stomach.

As bearing on this point the lecturer quoted the recent researches of Bunge, which go to prove that iron in an inorganic form is not assimilable at all, and that its usefulness in chlorosis is due to its neutralising the alkaline sulphides which decompose in the bowel the complex organic compounds of food which contain iron in an assimilable form, thus robbing the system of the supply of that element which it needs for the formation of hæmoglobin. This view illustrates two points: (1) Why aperients alone are so beneficial in chlorosis; (2) why iron in large doses does so much more good than in small ones.

